# Core health-components, contextual factors and program elements of community-based interventions in Southeast Asia – a realist synthesis regarding hypertension and diabetes

**DOI:** 10.1186/s12889-021-11244-3

**Published:** 2021-10-22

**Authors:** Zinzi E. Pardoel, Sijmen A. Reijneveld, Robert Lensink, Vitri Widyaningsih, Ari Probandari, Claire Stein, Giang Nguyen Hoang, Jaap A. R. Koot, Christine J. Fenenga, Maarten Postma, Johanna A. Landsman

**Affiliations:** 1 Department of Health Sciences, University of Groningen, University Medical Center Groningen, Hanzeplein 1, Building 3217, 9700 RB Groningen, The Netherlands; 2 Faculty of Economics and Business, University of Groningen, Groningen, The Netherlands; 3 Department of Public Health, Faculty of Medicine, Universitas Sebelas Maret, Surakarta, Indonesia; 4 HelpAge International, Yangon, Myanmar; 5 Health Strategy and Policy Institute, Hanoi, Vietnam; 6 Department of Pharmacology and Therapy, Faculty of Medicine, Universitas Airlangga, Surabaya, Indonesia; 7 Center of Excellence in Higher Education for Pharmaceutical Care Innovation, Universitas Padjadjaran, Bandung, Indonesia

**Keywords:** Community-based interventions, Southeast Asia, Non-communicable diseases, Diabetes, Hypertension, Core health-components, Contextual factors, Program elements, Realist synthesis/review

## Abstract

**Background:**

In Southeast Asia, diabetes and hypertension are on the rise and have become major causes of death. Community-based interventions can achieve the required behavioural change for better prevention. The aims of this review are 1) to assess the core health-components of community-based interventions and 2) to assess which contextual factors and program elements affect their impact in Southeast Asia.

**Methods:**

A realist review was conducted, combining empirical evidence with theoretical understanding. Documents published between 2009 and 2019 were systematically searched in PubMed/Medline, Web of Science, Cochrane Library, Google Scholar and PsycINFO and local databases. Documents were included if they reported on community-based interventions aimed at hypertension and/or diabetes in Southeast Asian context; and had a health-related outcome; and/or described contextual factors and/or program elements.

**Results:**

We retrieved 67 scientific documents and 12 grey literature documents. We identified twelve core health-components: community health workers, family support, educational activities, comprehensive programs, physical exercise, telehealth, peer support, empowerment, activities to achieve self-efficacy, lifestyle advice, activities aimed at establishing trust, and storytelling. In addition, we found ten contextual factors and program elements that may affect the impact: implementation problems, organized in groups, cultural sensitivity, synergy, access, family health/worker support, gender, involvement of stakeholders, and referral and education services when giving lifestyle advice.

**Conclusions:**

We identified a considerable number of core health-components, contextual influences and program elements of community-based interventions to improve diabetes and hypertension prevention. The main innovative outcomes were, that telehealth can substitute primary healthcare in rural areas, storytelling is a useful context-adaptable component, and comprehensive interventions can improve health-related outcomes. This extends the understanding of promising core health-components, including which elements and in what Southeast Asian context.

**Supplementary Information:**

The online version contains supplementary material available at 10.1186/s12889-021-11244-3.

## Background

Community-based interventions may have a positive effect on the prevention, cure, and care of non-communicable diseases (NCDs). In Southeast Asia, NCDs are the leading cause of death; it is estimated that 8.5 million deaths were due to NCDs, 48% of which occurred before the age of 70 years [1]. More specifically, diabetes and hypertension are continuously on the rise and have become a major public health issue [2]. In addition, genetic, acquired, environmental and societal risk factors of diabetes and hypertension are expected to increase [3]. This is due to reasons such as urbanization, low-income status, low awareness, upward trends in smoking, obesity, and alcohol use. Decreasing the burden of NCDs is difficult [4], mainly because environmental and economic factors are difficult to control and it requires behavioural changes in order to effectively reduce risk factors, which is not easy to achieve.

Influencing solidarity, self-reliance, and social support [5] make community-based interventions an effective way to induce behavioural change. Behavioural risk factors are shaped by societal conditions, and community-based interventions are aimed at and implemented in a population, therefore reaching individuals with varying levels of risk [6]. Community-based interventions refer to multi-component interventions that combine individual and environmental change strategies to prevent dysfunction and promote well-being among population groups in a defined local community [7]. According to Trickett et al. [8], community-based interventions are complex social processes, that go beyond single interventions and outcomes at individual levels of short-term change. Moreover, as community-based interventions are organized within different cultures, structures, and relationships [9], a particular intervention will only ‘work’ if the contextual conditions and certain program elements are conducive to its implementation. Based on this, we used a definition by combining those previously mentioned: Community-based interventions are complex social processes, that include multi-component interventions, aimed at preventing illness and unhealthy behaviour and promoting well-being among population groups in a defined local community in their context.

**Fig. 1:**
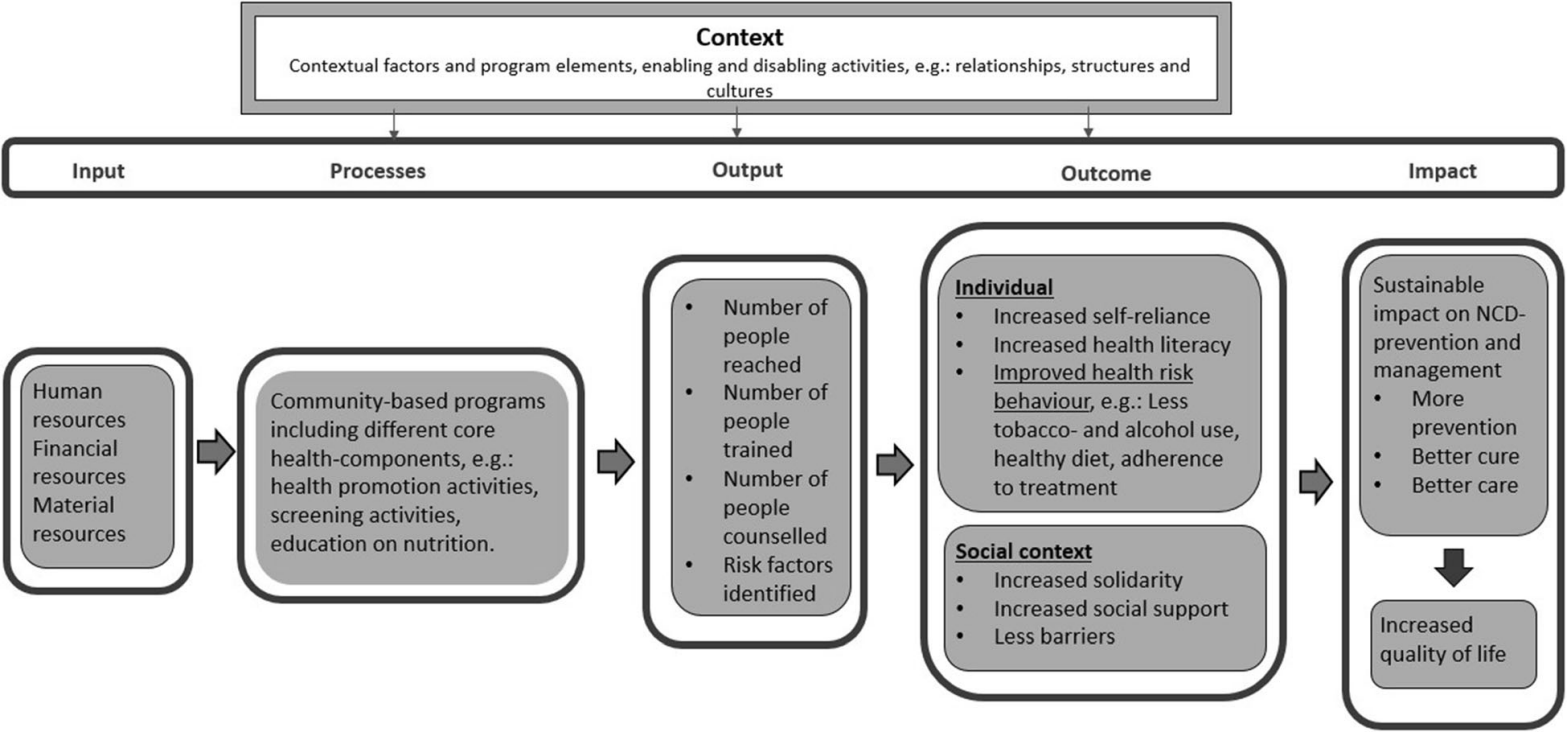
Framework of casual chain of community-based programs

In Southeast Asia, it is common practice to have community-based interventions targeting various aims, including NCD prevention [10]. Community-based interventions can encourage healthy behaviour, thus probably reducing health risk behaviour, which may have a sustainable impact on NCD-prevention and management. Figure 1 illustrates a framework, based on the WHO input-process-output-outcome-impact model [11], depicting theories of change of community-based programs aimed at NCD-prevention and management. Community-based programs need different resources (input), which enable core health-components and activities of these programs (processes), in turn resulting in products within these processes (output), which result in short and intermediate outcomes (outcomes), and eventually long-term outcomes (impact). In this review, we defined core health-components as ‘the essential functions or principles, associated actors and processes and intervention activities that are judged necessary to produce desired outcomes’, derived from a previous definition of Blase and Fixsen [12]. Moreover, this causal chain of input-processes-output-outcome-impact is organized within and affected by different environments (context) [13]. Within different environments, various conditions (contextual factors and program elements) indirectly enable or disable the intervention. With this framework, based on examples, we illustrate how we expect core health-components in community-based interventions, in certain contexts, to work. The framework was used for data extraction in this review. However, evidence on the link between community-based interventions and health is scattered. In addition, little is known about contextual influences and program elements; for instance, the synergy with health-facility-based NCD-interventions. Therefore, the aims of this review are, to extensively assess 1) the core health-components of community-based interventions and, 2) which contextual factors and program elements affect the impact of community-based interventions on health, in Southeast Asia. Core health-components are described as part of the processes, whereas the contextual factors and programs elements as part of the context. This is in line with the theory of change.

This study was performed in the context of an EU-Horizon 2020 funded project, ‘*Scaling up NCDs interventions in Southeast Asia’,* in which effective scaling up strategies of evidence-based diabetes and hypertension prevention and management programmes are researched, amongst others targeting Indonesia and Vietnam.

## Methods

### Design of the study realist review and context

We used a realist review design [5]. This is a method of systematically reviewing complex social interventions, like community-based interventions, based on the RAMESES (Realist and Meta-narrative Evidence Syntheses: Evolving Standards, see Additional file 1) [14]. A realist review is theory-driven, which involves identifying underlying causal mechanisms, and exploring how they work under different circumstances and contexts.

Scientific documents and grey literature in both the English language and local languages were included. Grey literature, i.e. not peer reviewed and not formally published in journals, was included to review aspects and settings closer to the communities and their context. For the grey literature, we selected two countries which can be considered representative of the Southeast Asian region and could be assessed thoroughly because of close collaboration in ‘Scaling up NCDs interventions in Southeast Asia’ project”, i.e. Indonesia and Vietnam. Combining empirical evidence with theoretical understanding, results in explanatory analysis about what could work, for whom and in what circumstances.

### Search method

We searched in the following databases: PubMed/Medline, Web of Science, Cochrane Library, Google Scholar and PsycINFO, in May 2019 with search terms such as ‘Southeast Asia’, ‘Community-based intervention’, ‘Diabetes’ and ‘Hypertension’ (for all search terms see Additional file 2) and a first selection based on title was done. Researchers in Indonesia and Vietnam completed a search in their own countries, in which the focus was on both offline printed materials and online resources, such as policy documents, evaluation reports, offline journals or power point presentations; mostly in the local language. The search resulted in a list of titles, numbered in an Excel sheet. After removal of the duplicates, we screened the abstracts.

We identified documents based on the following inclusion criteria: documents that report on community-based interventions/activities/programs aimed at the prevention, curing, or caring of diabetes and/or hypertension in Southeast Asian context; documents that report on health-related outcome, and/or impact, and/or output of community programs, for instance: quality of life or burden of NCDs; documents describing contextual factors and/or program elements affecting the characteristics and core health-components in community-based interventions, for instance: gender and accessibility; documents published between 2009 and 2019.

Multiple collaborating researchers (full names and affiliations in the acknowledgements) systematically described reasons for in- and exclusion of selected scientific documents and grey literature. ZEP and JAL checked the quality by reassessing a random sample of three selected documents. Subsequently, all documents were systematically screened and, of those included, data was captured in the data-extraction form. The data-extraction form (available from first author) was used for recording information per study, regarding general information, characteristics of the included population, general description of intervention, and measures of input, output, outcome and impact. Regarding the outcome, we defined effectiveness as that health components improve output and outcome, and thus, potentially also impact. Furthermore, they reported whether contextual factors and program elements affected the effectiveness of these core health-components. The data-extraction form was developed using theory of change*.* The data were then analyzed, resulting in an overview of detailed information. The data were synthesized based on the findings for context, input, processes, output, outcome, and impact. The findings were converted to the core health-components, possible contextual factors, and program elements.

### Quality assessment

We assessed quality of both scientific and grey literature using three study-type specific tools: Firstly, *the QUIPS Risk of Bias Assessment Instrument for Prognostic factor studies* [15] for quantitative studies. This rating instrument assesses the potential bias to be low (3 points), moderate (2 points) or high (1 point) for six domains: study participation, study attrition, prognostic factor measurement, outcome measurement, study confounding and statistical analysis and reporting. Secondly, we used the *Quality assessment of qualitative evidence tools* [16] for qualitative studies, in which ratings of good (4 points), fair (3 points), poor (2 points) and very poor (1 point) are given to following domains: abstract and title, introduction and aims, method and data, sampling, data analysis, ethics and bias, results, transferability or generalizability, and implications and usefulness. Thirdly, for reviews we used the *Critical Appraisal for Systematic reviews from Centre for Evidence-Based Medicine* [17], in which ratings of yes (1 points), no (0 points) or unclear (0 points) were giving to the following domains: clear question addressed in the review; unlikeliness that relevant studies were missed; appropriate criteria for selection of the articles; sufficiently valid included studies for type of question asked; and similar results found in studies. The ratings of the three quality assessment tools were combined into one scale, classifying three categories, namely: 1 = high quality (quantitative studies scoring 15–18 points; qualitative studies scoring 30–36 points; and reviews scoring 4 or 5 points), 2 = moderate quality (quantitative studies scoring 7–14 points; qualitative studies scoring 24–29 points; and reviews scoring 2 or 3 points) and 3 = low quality (quantitative studies scoring 6 points or lower; qualitative studies scoring 9–23 points; and reviews scoring 0 or 1 point).

### Data analysis and reporting

Firstly, we gave an overview of the characteristics of the documents. Secondly, we described the core health-components, and the contextual factors and program elements affecting the impact of the core health-components. We did this separately for the scientific and grey literature, to determine the added value of the grey literature to the scientific literature.

## Results

### General description of the documents

**Fig. 2:**
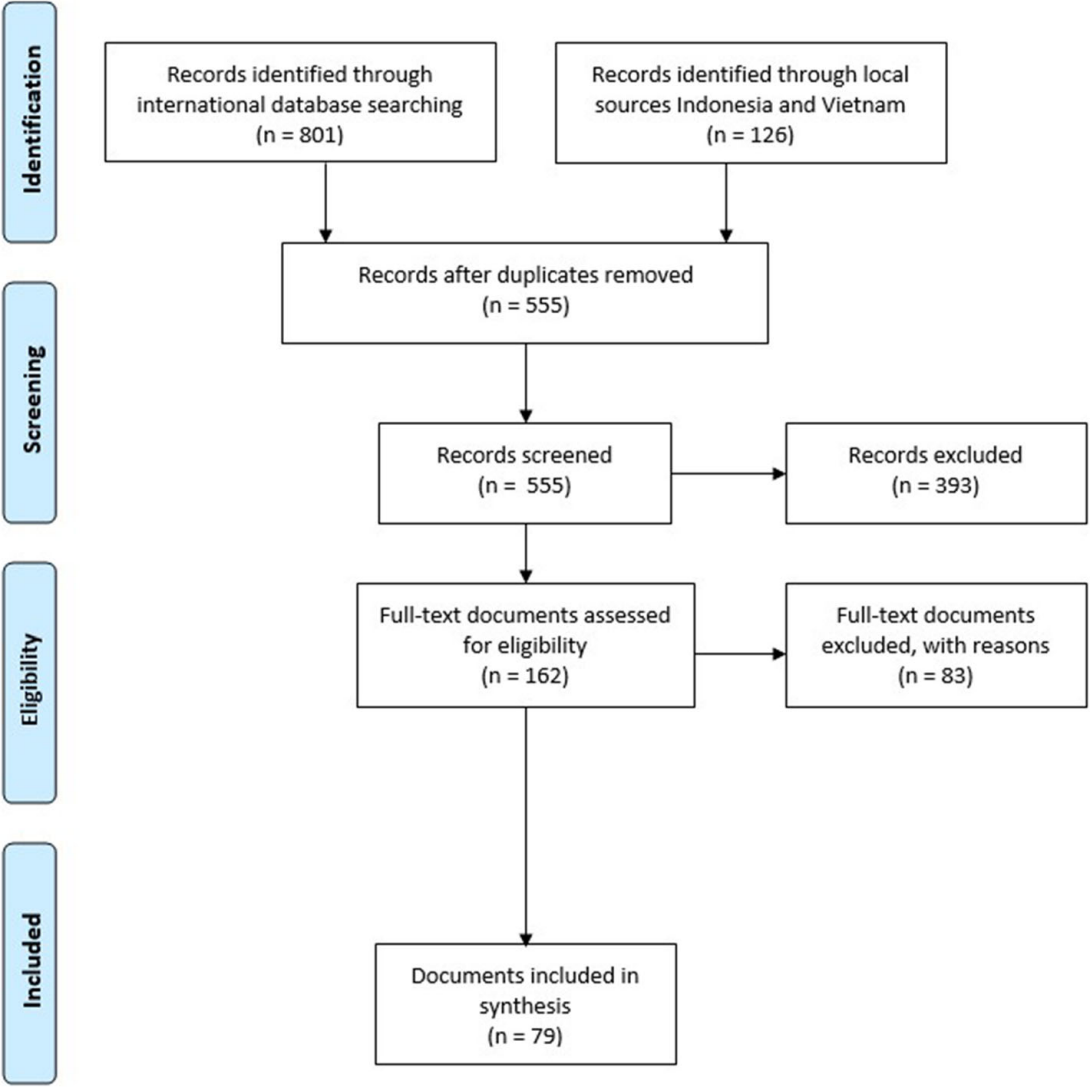
Identification of documents based on PRISMA flow diagram [114]

After removal of the duplicates, the search resulted in 555 titles. After screening the titles and abstracts, 162 documents remained and we selected 79 relevant documents (see Fig. 2).

Of the 79 documents, 20 came from the international search, 50 from the Indonesian search and nine from the Vietnamese search (see Table 1). The countries included in the documents were Cambodia, Indonesia, Malaysia, The Philippines, Thailand and Vietnam. Sixty-seven documents were published in the scientific literature and 12 documents in the grey literature. In the scientific literature, three documents were systematic reviews, 28 documents had a descriptive quantitative or qualitative design, 12 documents had a quasi-experimental design, 19 documents had a cross-sectional design, three documents were randomized controlled trials, and two documents had a longitudinal design. The grey literature regarded five theses, six (policy-)reports and one PowerPoint presentation. Of these, three studies had a quasi-experimental design, one had an observational design, two were literature reviews, three had a descriptive qualitative design, two had a descriptive quantitative design, and one had a cross-sectional design.

**Table 1:** Distribution of type of disease/risk factor per document and divided in country searches

Type of disease/risk-factor	Number of documents	Indonesia	Vietnam	International search
Diabetes	**23**	8	1	14
Hypertension	**24**	16	5	3
Diabetes & hypertension	**4**	–	2	2
NCDs in general	**28**	26	1	1
Total	79	50	9	20

The majority of the documents focused on interventions aimed solely at either diabetes or hypertension, or interventions aimed at multiple NCDs (Table 1). Others included documents focused on both diabetes and hypertension. Most documents described interventions such as health education, health behavior, apps/websites, supply of tools or health promotion.

Twelve documents were classified as having low quality, 52 as having moderate quality and 15 as having high quality. The 67 scientific documents were all peer-reviewed. Twenty-two documents were in the English language and 57 documents were in local languages. All the documents were included in the review. The main findings were based on high quality documents and strengthened by those with moderate and low quality.

Table 2, Additional file 3, presents the main characteristics, the core health-components, the contextual factors, and program elements. Fifty-nine documents addressed core health-components and 20 addressed contextual factors and program elements.

#### Core health-components

**Fig. 3:**
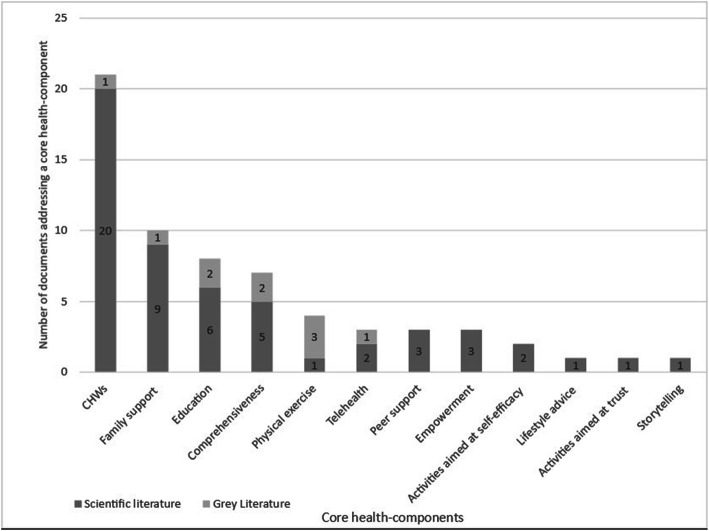
Number of documents reporting on core health-components in scientific and grey literature

We found 12 core health-components of interventions. Figure 3 shows the division of the core health-components described in the scientific or the grey literature. The core health-component *Community Health Workers (CHWs)* is most commonly described, namely in 20 scientific documents and one grey literature document. *Lifestyle advice*, s*torytelling* and *activities aimed at establishing trust* are described the least, namely, all three are mentioned once in three separate scientific documents. All core health-components were described in the scientific literature, and six core health-components were also found in the grey literature. Most documents, 48 scientific and nine grey, described one core health-component and five documents described multiple core health-components. The results are described below in the scientific literature and grey literature sections.

### Core health-components described in the scientific literature

*Community Health Workers* (CHWs), i.e. members of communities, selected by communities and trained to carry out functions related to healthcare, can function as gatekeepers between primary healthcare and community members [24, 85]. CHWs can help with adherence to treatment in rural communities by giving social support [24, 25]. Several documents reported that knowledge and skills of CHWs can be improved by comprehensive health education programs, training in communication, and usage of equipment and involvement of different stakeholders (for example primary healthcare staff) [21–23, 26–36, 41, 66, 72, 80, 86–88]. Related to this, support given by CHWs to community members, was the most dominant factor in the utilization of community-based interventions by community members [41].

Several documents reported a positive correlation of *family support* with patient behaviour and adherence to treatment that next affected management and control of NCDs [37, 39–44, 52, 89, 90]. In these cases, families were involved in the intervention and their knowledge and attitude towards NCDs improved.

Furthermore, the element of E*ducation* in which the knowledge of community members increases, improved health-related behaviour [18, 51, 63, 69], self-efficacy [53, 91], health literacy [54], and decreased levels of risk factors for NCDs [56].

*Comprehensive* community-based interventions, consisting of multiple elements, such as a combination of physical exercise and a nutrition program [66, 67], education and physical exercise [69] or guidance and education [64], were determined to improve knowledge about diseases, by providing access to information about health, and health-related behaviour.

*Physical exercise* was found reduce the risk factors of NCDs [60] and *telehealth*, i.e. engaging people via phone, computer, online, or social media, can bridge the communication gap between patients and the health system, by connecting community members with health professionals [61, 92].

Another core-element that was utilized for the prevention, cure and care of NCDs, is *peer support* [24, 44–46], i.e. the provision of emotional and informational assistance by a person within the social network, who has knowledge of a specific behavior or stressor and similar characteristics as the target population, to address a health-related issue of a potentially or actual stressed focal person [93]. Peer support was found to be an important motivator for the participation in community-based programs and helps increase the quality of care for those with diabetes and hypertension. Peers provided care to a large number of patients in rural areas by reducing existing barriers, increasing access to diagnosis and treatment and providing disease education and support [24, 46].

Furthermore, multiple documents showed that *empowerment* of community members, by education to improve knowledge and the effect of health campaigns, was a core health-component of community-based interventions [19, 20, 55]. Likewise, *activities to achieve self-efficacy*, which is defined as the belief in one’s own ability to meet challenges and complete tasks successfully, e.g. performance accomplishment and vicarious experiences, targeting diabetes, have an influence in enhancing patient’s health [48, 91].

Other core health-components found were lifestyle advice from a community pharmacist, which increased the treatment adherence of patients with diabetes [50, 94], the establishment of empowerment programs and *activities aimed at establishing trust*, e.g. facilitating respect and listening to each other, between community members and professionals, village or community workers, is important [47].

Allison et al. [49] showed that in rural areas of Vietnam, education, by *storytelling* about one’s own experiences with NCDs, was an effective component in community-based interventions.

### Additional core health-components described in grey literature

Multiple components, found in the scientific literature, were also mentioned in the grey literature; namely, appointing CHWs, family support, comprehensiveness, *physical exercise, telehealth and education* [38, 51, 57–59, 62, 65, 68]. Hung [62] also reported that *telehealth*, by providing online communication tools on a website, promotes communication between patients and the health system in rural areas. Truong et al. [65] reported that communication with community members who are NCD patients, was an important component of the *education* for CHWs.

#### Contextual factors and program elements

**Fig. 4:**
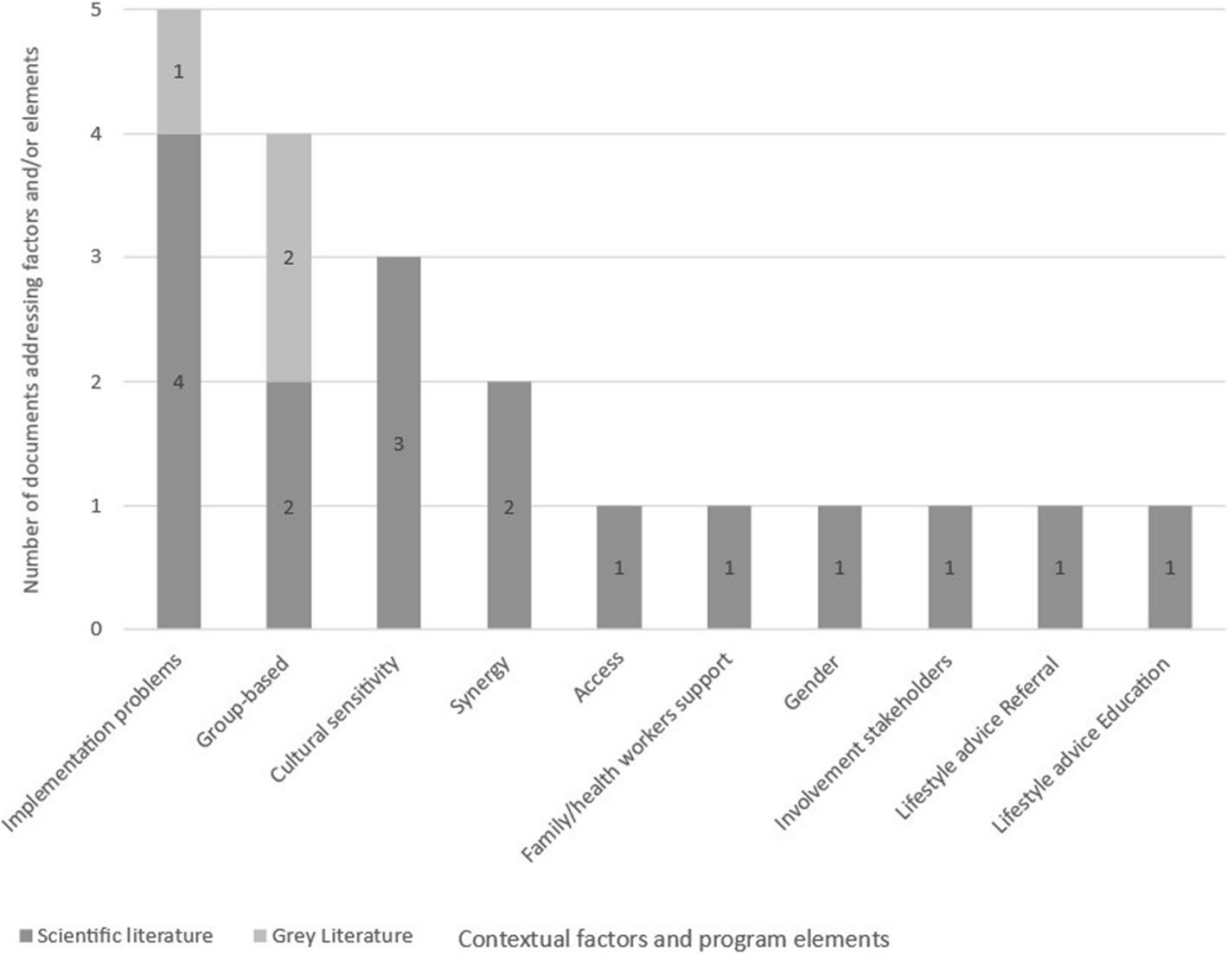
Number of documents reporting on factors and elements in scientific and grey literature

Regarding the second aim of this research, i.e. assessing which contextual factors and program elements affect the impact of community-based interventions on health, 10 contextual factors and program elements were identified. In Fig. 4, the contextual factors and program elements are displayed, split into two categories: scientific and grey literature. *Problems with implementation* are described in most documents. Most factors and elements are described in only one scientific document. Further findings were reported separately for the scientific and the grey literature.

### Contextual factors and program elements in the scientific literature

In Indonesia, *implementation problems* are the most mentioned contextual factors. This, for example, concerns insufficient human resources, incomprehensive and minimal training, insufficient equipment, financial dependence, no clear role of CHWs, and no coordination of activities in the program [71–73, 95].

Moreover, multiple documents reported that when activities in community-based interventions were organized *group-based*, people encourage each other in improving health-related behaviour [81, 84].

Multiple documents reported finding evidence for the influence of the contextual factor *cultural sensitivity* on the effects of community-based interventions [70, 96, 97]. When community-based interventions are tailored to the local language, including the lingo, custom traditions and adapted to preferences, needs, values, interests, religion, and other sociocultural specific aspects, trust, comprehensibility and motivation among participants raise, resulting in improved health-related outcomes.

Furthermore, we found that, to increase the participation of community members, a better *synergy* is needed between primary healthcare facilities and communities [76, 77]. One of the barriers in participation is when there are no drugs available at the community-level or when community members must pay for the drugs themselves.

Next, *access, family/health workers support, and gender* were coherent with the utilization of community programs in Indonesia [75, 78, 98]. When access to the program was more difficult (i.e. pay a fee or the location is difficult to reach), utilization was lower. On top of this, when family or CHWs support was high, utilization of programs was higher. Moreover, more women than men participated in programs, probably because these programs were organized during working hours.

Moreover, a program element that increased the impact of telehealth *is involving different stakeholders*, such as a clinical team [61]. The major difficulty with telehealth, specifically in geographically and socially disadvantaged populations, is access to these technologies, which is often limited or absent [61].

The program elements affecting lifestyle advice from community pharmacists are *referral and education services* [94]^.^ In the case of rural communities, referral, and access to a hospital and/or primary healthcare facility can be difficult, if not impossible. Besides this, when there is a primary healthcare facility in a rural community, the knowledge of primary healthcare staff about the importance of a community-based intervention as a means to meet health needs, was often not sufficient and therefore, sustainable *education* for health staff was needed [79].

### Additional contextual factors and program elements in grey literature

In line with the scientific literature, we found *implementation problems* with the community programs in Indonesia, such as insufficient human resources, minimal training, lack of equipment and financial dependence [74].

Moreover, in correspondence to the scientific literature, Hanh [82] and Long [83] also found that community-based interventions, when organized in *groups,* results in higher degree of participation due to increased encouragement by community members.

## Discussion

We found twelve core health-components of community-based interventions, i.e. actors and processes directly related to actions in health interventions, and ten contextual factors and program elements, i.e. conditions that enable or disable the intervention directly. Most described core health-components were: CHWs, family support and education. The most innovative components we found were: comprehensiveness, telehealth and storytelling. Implementation problems, group-based organized and cultural sensitivity are most mentioned as influencing cultural factors and program elements. Our findings were fairly robust and generally remained consistent, if restricted to the documents with high quality. The results of this review align with expectations based on the theory of change. Below, we interpret our findings on the core health-components, contextual factors, and intervention elements, building further on the theoretical framework described in the introduction.

We found that comprehensiveness in community-based interventions, including multiple strategies, such as physical exercise and influencing self-efficacy, improves health-related outcomes. This corresponds with evidence from previous reviews [99, 100] that reported that interventions with multiple strategies were more likely to be effective. This includes a higher number of activities, targeting various aims, seems to have a higher impact on diabetes and hypertension prevention and management. Even more effective, next to comprehensive activities, is comprehensive targeting in an intervention [101]. Geboers et al. [101] found that comprehensive targeting in interventions improves health outcomes, especially for people with low health literacy. Interventions should target (1) individuals’ personal characteristics, (2) individuals’ social context (3) communication between individuals and health professionals, (4) health professionals’ health literacy capacities and (5) the health system. This corresponds to findings of the following components, factors and elements: family and peer support, involvement of different stakeholders and creating synergy between community programs and primary healthcare.

One of the innovative findings is that telehealth is a core health-component of community-based interventions, especially in rural areas. However, we also found that telehealth is more difficult to establish in more remote and rural areas, because access to technology and internet connection is often limited, absent, or only available under specific conditions [61]. Multiple studies have shown that telehealth can improve patient care and health outcomes, yet these findings were mostly based on urban areas [102–104]. In rural areas with limited access to healthcare facilities, telehealth can substitute certain healthcare needs of community members, if the technical infrastructure allows for it. Multiple articles described how to overcome these technical difficulties in rural areas, for example, by providing stable internet access for patients and providers, providing telehealth devices, and providing education about the benefits and use of telehealth [105–108]. Moreover, the rapid expansion of the global telecommunications network may solve the problem in due time [109].

Another of the innovative findings, regarding core health-components, we found is storytelling. Research in health literacy programs showed that, especially for people with low literacy skills who struggle to read and understand written health information, non-written strategies of storytelling can increase health literacy [110]. Koops van ‘t Jagt et al. [110] revealed that providing narrative forms of health communication, i.e. photo novellas, video and live storytelling, are promising empowering strategies for improving health. Moreover, this component is naturally adapted to the context, making it culturally sensitive, and likely improving health-related outcomes given the results of our review. Folklore, i.e. traditional beliefs, customs, and stories of community, passed through the generations by word of mouth, is a common way of sharing information in Asia [111]. Therefore, we expect that storytelling can be a promising core health-component in community-based interventions.

Regarding contextual factors, we found multiple documents that reported implementation problems as potential barriers, such as insufficient equipment and human resources. Implementation problems can be the result of diminished implementation fidelity, i.e. the extent to which an intervention is delivered as intended [112]. According to Breitenstein et al. [112], implementation fidelity in community-based interventions is often low, because they are not adapted to real life contexts and culture. This corresponds with our finding that tailoring community-based interventions to the culture, improves health-related outcomes. To increase implementation fidelity, one might consider tailoring interventions to culture by including local adaptions*,* such as local perceptions on health, and myths and facts regarding health promotion.

### Strengths and limitations

This study has some major strengths, particularly, the comprehensiveness of its searches, including grey and local language literature and various research designs. This includes context and elements, therefore including non-controlled settings, provides conclusions that we can learn about real world settings [9].

An important limitation of this study is that the quality measures may have been less suitable for the grey literature. For the quality assessment of this review, three instruments, developed for assessing the quality of scientific literature, were used. This resulted in the assessment of most grey literature as low quality. Moreover, most grey literature included, had designs which could only provide weak evidence regarding effectiveness, e.g. due to lack of a control group. The grey literature is considered as important additional information, which contributes to in-depth conclusions. Quality instrument could be developed further in this regard.

Another limitation is the heterogeneity in the use of terminology in different languages. Translation from English to local languages and vice versa, could have led to ambiguity in the search and data extraction.

One more limitation of this study is that several conclusions are supported by a small number of studies, occasionally only one to three studies. These conclusions evidently require a further strengthening of their empirical support.

### Implications

We found that comprehensive community-based interventions have a larger impact on diabetes and hypertension, which implies that future community-based intervention could best include multiple components that address various targets that include the social and healthcare context. Including various targets could be completed by the core health-components family and peer support. Another way is involving various target levels, e.g. healthcare professionals to increase synergy. This is expected to have a higher impact on prevention, cure and care of diabetes and hypertension.

Moreover, we found telehealth as potential component of community-based health interventions, especially in rural areas where healthcare facilities are limited. This could be elaborated further, as technology and internet connections are evolving rapidly, even in rural areas, for prevention of and care for NCDs.

Finally, we found storytelling to be a promising core health-component of community-based interventions. Involving peers that people with NCDs can relate to, especially when health literacy is low, can be a context-adapted component of interventions. More research might consider the outcomes and effectiveness of telehealth, storytelling and several contextual factors, i.e. cultural sensitivity in Southeast Asia, as evidence on these approaches is limited to one to three studies. Moreover, research into the costs and benefits of components and factors could provide a stronger empirical basis for efficacious implementation. Further research is thus needed to strengthen the evidence on these core health-components, contextual factors, and intervention elements.

For all included countries, we found at least one study about education, for community members, CHWs or peers. This suggests that education is an important component of community-based programs for prevention, cure and care of NCDs in Southeast Asia. This could reflect the acknowledgement of average low levels of health literacy in Southeast Asia [113], thus requiring interventions to improve health literacy in the region.

Regarding research, our study shows that inclusion of grey literature can contribute to studying effectiveness of the core health-components of community-based interventions. Future reviews that include grey literature might consider carefully consider how to assess quality of non-scientific documents.

## Conclusions

Our review provides an overview of core health-components and contextual factors and program elements of community-based interventions, regarding diabetes and hypertension in Southeast Asia. We found a number of core health-components that strengthen community-based interventions: community health workers, family support, education, comprehensiveness, physical exercise, telehealth, peer support, empowerment, activities to achieve self-efficacy, lifestyle advice, activities aimed at establishing trust and storytelling. In addition, we found the following contextual factors and program elements that may affect the impact: implementation problems, group-based organized, cultural sensitivity, synergy, access, family health/worker support, gender, involvement of stakeholders and referral and education services when giving a lifestyle advice. By using a realist methodology, this review contributes to an in-depth understanding of what core health-components work in community-based interventions, including which factors and elements, in what Southeast Asian context.

## Supplementary Information

**Additional file 1.** Table with RAMESES checklist.

**Additional file 2.** Search terms.

**Additional file 3.** Table with study characteristics.

## Data Availability

N/A

## Abbreviations

e.g.: Exempli gratia, Latin phrase meaning “for example”
CHW: Community health worker
i.e.: Id est., Latin phrase meaning “that is”
NCD: Non-communicable disease
WHO: World Health Organization
