# Supplementary material for: Core health-components, contextual factors and program elements of community-based interventions in Southeast Asia – a realist synthesis regarding hypertension and diabetes
Source: BMC Public Health. 2021 Oct 22;21:1917. doi: 10.1186/s12889-021-11244-3 (PMC8539840; doi:10.1186/s12889-021-11244-3)
Supplement: Supplementary file 2 — Additional file 2. Search terms. [file 12889_2021_11244_MOESM2_ESM.docx]

#### Additional file 2: Search terms

*Southeast Asia[Mesh terms] OR southeast Asia[Title/Abstract] OR Southeast Asia[Title/Abstract] OR Indonesia[Title/Abstract] OR Myanmar[Title/Abstract] OR Vietnam[Title/Abstract]*

AND

*Diabetes[MeSH Terms] OR Diabetes[Title/Abstract] OR Diabetes Mellitus [Title/Abstract] OR Prediabetes[Title/Abstract] OR hyperglycemia[Title/Abstract] OR hyperglycemia[Title/Abstract] OR High blood glucose[Title/Abstract] OR Type 2 diabetes[Title/Abstract] OR metabolic syndrome[Title/Abstract]*

AND/OR

*Hypertension[MeSH Terms] OR Hypertension[Title/Abstract] OR hypertensive [Title/Abstract] OR blood pressure [Title/Abstract] OR high blood pressure [Title/Abstract] OR prehypertension [Title/Abstract] OR raised blood pressure*

AND

*Community[MeSH Terms] OR Community[Title/Abstract] OR Community group[Title/Abstract] OR Communities[Title/Abstract] OR community group[Title/Abstract] OR community program[Title/Abstract] OR community programs[Title/Abstract] OR community approach[Title/Abstract] OR community participation[Title/Abstract] OR Target group[Title/Abstract] OR Target groups[Title/Abstract] Or Program[Title/Abstract] OR Community-based[Title/Abstract] OR Community Intervention[Title/Abstract]*

AND

*Intervention[Title/Abstract] OR health promotion[Title/Abstract] OR health awareness[Title/Abstract] OR Health Education[MeSH Terms] OR health activities[Title/Abstract] OR Self-management[Title/Abstract] OR health literacy[Title/Abstract] OR adherence[Title/Abstract] OR lifestyle change[Title/Abstract] OR lifestyle changes[Title/Abstract] OR health behavioural change[Title/Abstract] OR health behaviour change[Title/Abstract] OR health behaviour change [Title/Abstract] health behavioural change[Title/Abstract] or dietary changes[Title/Abstract] OR dietary change[Title/Abstract] OR diet change[Title/Abstract] OR diet changes[Title/Abstract] OR physical exercise[Title/Abstract] or health knowledge[Title/Abstract] OR health skills[Title/Abstract] OR Quality of life[Title/Abstract] OR Control[Title/Abstract] OR Treatment[Title/Abstract] OR Prevention[Title/Abstract] OR Education[Title/Abstract] OR Empowering [Title/Abstract] OR Effectiveness[Title/Abstract] OR Impact[Title/Abstract] OR Practice[Title/Abstract] OR Practices[Title/Abstract] OR Care[Title/Prevention] OR Cure[Title/Prevention]*
