# Supplementary material for: Core health-components, contextual factors and program elements of community-based interventions in Southeast Asia – a realist synthesis regarding hypertension and diabetes
Source: BMC Public Health. 2021 Oct 22;21:1917. doi: 10.1186/s12889-021-11244-3 (PMC8539840; doi:10.1186/s12889-021-11244-3)
Supplement: Supplementary file 3 — Additional file 3. Table with study characteristics. [file 12889_2021_11244_MOESM3_ESM.docx]

#### Additional file 3: Table with study characteristics

Main characteristics, Core health-components and contextual factors or program element

| **Study** | **S/G^*^** | **U/R^**^** | **Quality level^***^** | **Country^****^** | **Intervention** | **Core health components and/or contextual factors and program elements** |
| --- | --- | --- | --- | --- | --- | --- |
| Carolina, 2018 | S | R | 2 | I | Health education about diabetes | **Empowerment** by improving knowledge of community members through education is effective in improving healthy lifestyle |
| Sukesi, 2019 | S | R | 2 | I | Counseling via PowerPoint and leaflets | **Empowering** community members by improving knowledge can improve healthy behaviour and prevention |
| Triyanto et al., 2015 | S | R | 2 | I | Empowerment model to increase adherence of diabetes patients | Diabetes patients can be **empowered** by education in self-care and improvement of knowledge |
| Trisnowati, 2018 | S | R | 2 | I | Empowerment of CHWs | Empowerment of **CHWs** is effective for prevention of risk factors of NCDs |
| Mokoginta & Palandeng, 2016 | S | U | 2 | I | Knowledge of CHWs | Involvement of different stakeholders, i.e. healthcare staff, in training will improve knowledge and attitude of **CHWs** |
| Indarjo et al., 2019 | S | R | 3 | I | Increasing knowledge and skills of CHWs | **Empowerment** of **CHWS** by improving knowledge by multiple education programs. |
| Maulida et al., 2015 | S | NC | 2 | I | Community NCD program | Communication is an important component of education for **CHWs** |
| Nursiswati et al., 2013 | S | R | 2 | I | Improving knowledge and abilities of CHWs | Education for **CHWs** is effective in improving knowledge and skills in self-care and management of diabetes. |
| Rahmawati & Bajorek. 2015 | S | R | 2 | I | community-based program in supporting and screening patients with hypertension | **CHWs** are gatekeepers between primary healthcare and rural communities and are especially effective when giving social support to the community. **Peer support** is effective for NCDs prevention. |
| Istifada & Rekawati, 2019 | S | - | 2 | I | CHWs involvement in prevention of hypertension | **CHWs** link community and healthcare, are effective in preventing complications, help with adherence to treatment by giving social support to community |
| Fatmah, 2013 | S | U | 2 | I | Training to improve CHWs knowledge and skills of obesity and hypertension | **CHWs** knowledge and skills should be improved by multiple education programs and training |
| Pratiwi, 2017 | G | R | 3 | I | Empowerment program for CHWs | **CHWs** empowerment can improve skills and knowledge |
| Pranandari et al., 2017 | S | R | 2 | I | Community NCD program | **CHWs** support seems to affect utilization of a community-based program |
| Wagner et al., 2015 | S | R | 2 | C | Diabetes prevention curriculum | **CHWs** are effective when knowledge improves by education |
| Sranacharoenpong,& Hanning, 2011 | S | R&U | 1 | T | Training on diabetes for CHWs | **CHWs** training can improve knowledge |
| Sari & Yamin, 2018 | S | U | 2 | I | Education about early detection and self-care of diabetes for CHWs | Education of **CHWs** is effective in improving knowledge and skills |
| Nugraheni & Hartono, 2018 | S | U | 2 | I | Education of CHWs | Education of **CHWs** is effective in improving knowledge and skills |
| Fatmah, 2012 | S | U | 2 | I | Skills training and balanced nutrition counseling about hypertension for CHWSs | Education for **CHWs** can improve skills and knowledge |
| Arfianti, 2019 | S | R | 2 | I | Training to improve CHWs skills and knowledge about diabetes | Training **CHWs** is effective in achieving competency, knowledge and skills |
| Mardiah et al., 2015 | S | R | 2 | I | Empowerment of CHWs | Empowering **CHWs** is effective in prevention of hypertension |
| Sari et al., 2018 | S | R | 2 | I | Health kits with training for CHWs | **CHWs** support is effective in improving knowledge and management of hypertension |
| Restuastuti & Restila, 2018 | S | R | 2 | I | Training for CHWS on NCDs | **CHWs** training can increase knowledge |
| Setiyaningsih & Ningsih, 2019 | S | R | 2 | I | Motivation, family and CHWs support to control hypertension | **Family support** and **CHWs** affect motivation on hypertension control behaviour |
| Wicaksono & Alfianto, 2019 | G | R | 3 | I | Community NCD program | **Family support** affects control of hypertension by patients |
| Tarigan et al., 2018 | S | R | 2 | I | Community NCD program | **Family support** is effective in encouraging healthy behaviour and improving knowledge of hypertension |
| Nurhidayat, 2016 | S | R | 2 | I | Involvement of family of hypertension patients | In rural communities, **family** involvement affects healthy lifestyle by controlling risk factors of hypertension |
| Umayana & Cahyati ,2015 | S | U | 2 | I | Relationship between family support and community leaders | Correlation between **family support** and community leader support |
| Herlinah et al., 2013 | S | U | 2 | I | Family support and control behaviour | There is a relationship between **family support** and control of hypertension behaviour |
| Ardian, 2013 | S | R | 2 | I | Improving knowledge of family | Improving knowledge and attitude of **family** members affects management and control of NCDs |
| Gumarang & Gita, 2015 | S | U | 2 | I | Nutrition program for hypertension | Level of knowledge of **family** members is in line with attitude towards diet behaviour by hypertension patients |
| Bratanegara et al., 2012 | S | U | 2 | I | Family support/ network support improvement | **Family support** and **peer support** have a correlation with patient behaviour and adherence |
| Marwati et al., 2017 | S | U | 2 | I | Improving family and member access to comprehensive health services | Involvement of **family** in community-based interventions is effective in increasing healthy behaviour |
| Paz-Pacheco. et al.,2017 | S | R | 1 | P | Education program for peer educators | **Peer educators**, giving advice and having group discussion can affect diabetes |
| Taniguchi et al., 2017 | S | R | 2 | C | Peer educators screening from house-to-house in each village for diabetes | **Peer educators** can provide support and self-management of patients both in individual as group setting |
| Suntayakorn & Rojjanasrirat, 2013 | S | R | 2 | T | Empowerment program to change unhealthy behavior | **Trust** is an important element to create empowerment in communities |
| Ahmad Sharoni et al., 2018 | S | NC | 1 | M | Health education programs based on the self-efficacy theory on foot self-care behaviour | **Self-efficacy** is an effective component of education in diabetes |
| Nguyen et al., 2017 | S | U | 1 | V | A self-efficacy theory based foot care education intervention program | **Self-efficacy** is an effective component of education to empower members |
| Allison et al., 2016 | S | R | 1 | V | Culturally adaptive storytelling intervention versus didactic intervention to improve hypertension control | **Storytelling** can improve hypertension control in rural areas |
| Pongwecharak & Treeranurat, 2011 | S | NC | 2 | M | Model for community pharmacists to screen and recommend lifestyle changes for diabetes patients | **Lifestyle advice** from a community pharmacist is effective in lifestyle changes. In combination with a risk assessment tool a lifestyle advice is effective, to **refer** patients |
| Fadila & Sutardi, 2014 | G | U | 2 | I | Health promotion through education | **Education** on nutrition can improve knowledge, attitude and practice in NCDs |
| Sari et al., 2018 | S | U | 2 | I | Family-Based Self-Management Education Group | **Education** in families is effective in improving knowledge and healthy behaviour |
| Nuraeni et al., 2017 | S | R | 2 | I | Multiple forms of education | Using multiple **education** components improves knowledge about self-efficacy in prevention and treatment of hypertension |
| Andrianys et al., 2017 | S | R | 2 | I | Education model to increase health literacy | **Education** can improve health literacy in a community |
| Sutini & Emaliyawati, 2018 | S | R | 2 | I | Education to empower community | **Education** to empower the community how to treat hypertension is effective with stress management |
| Hoa, 2015 | S | R | 1 | V | Health education intervention | **Education** is effective in decreasing risk factors in a community |
| Thang, 2017 | G | R | 3 | V | Training for community staff in communication on health education and control of hypertension | **Education** in communication about hypertension is effective |
| Anggraini, 2015 | G | R | 3 | I | Aerobic for hypertension patients | **Physical exercise** is effective in control of hypertension |
| Putri et al, 2015 | G | R | 3 | I | Exercise intervention | **Physical exercise** is effective in control of hypertension |
| Nguyen & Kruise, 2012 | S | U | 1 | V | Thai-Chi training program | **Physical exercise** is an effective community-based intervention |
| Ruddock et al., 2016 | S | R | 2 | SEA | Different strategies of telehealth | **Telehealth** is effective in connecting community members with primary healthcare, especially in **rural areas**. Yet, connection is often poor in rural areas |
| Hung, 2019 | G | R&U | 3 | V | Activities including online and offline at hospitals | **Telehealth** can be effective in promoting communication between patients and the health system in rural areas, increase awareness, early detection and improves the treatment quality |
| Mashitah, 2012 | S | R | 2 | I | Telehealth in participation and management | **Telehealth** is effective in the management of diabetes, by increasing knowledge, management and initiate physical activity. Specifically **in rural communities** |
| Oba, 2011 | S | NC | 2 | T | Health promotion program composed of nutritional education and exercise activity | Improving knowledge by **education** in combination with p**hysical exercise** improves a healthy lifestyle (**Comprehensive)** |
| Ratnawati et al., 2015 | S | R | 2 | I | Therapy, guidance, self-help groups, health education and coaching | Community-based intervention is effective when they consist of education and guidance (**Comprehensive**) |
| Truong et al., 2016 | G | R | 3 | V | Nutrition education model on hypertension | **Comprehensive** education in communication about nutrition and physical exercise has positive impact on knowledge and practice of hypertension |
| Tran et al., 2016 | S | U | 1 | V | Nutrition and exercise intervention | **Comprehensive c**ommunity-based interventions including nutrition education and exercise is effective |
| Tran et al., 2017 | S | R | 1 | V | Physical activity and nutrition program for diabetes patients | Community-based intervention is more effective when **comprehensive**, namely physical activity and nutrition program |
| Lanh, 2014 | G | R | 3 | V | Program to Increase knowledge about disease diabetes prevention | Community intervention about diabetes is effective when **comprehensive** with positive healthy activities and regular |
| Norris et al., 2014 | S | U | 2 | M | Multiple strategies and  resources to encourage participation and to improve retention | **Comprehensive** community interventions, including education and physical exercise are effective |
| Savitri & Sari, 2018 | S | U | 2 | I | Community NCD program | **CHWs support** is a dominant factor for utilization of a community program. |
| Gyawali et al., 2018 | S | R&U | 2 | SEA | Different interventions tailored to culture | **Cultural sensitivity** is important in community-based interventions |
| Niazi & Kalra, 2012 | S | NC | 1 | SEA | Sensitive model towards the patients' preferences, needs and values | Interventions need to be adapted to preferences, needs, values, interests, religion and **cultural aspects** |
| Ku & Kegels, 2018 | S | R & U | 1 | P | Context-adapted Chronic care mod | Adapting community-based interventions **to context** is needed |
| Putri et al., 2018 | S | R | 2 | I | Integrated community program for NCDs | **Implementation problems**, i.e. lack of volunteers, coordination, and sub-optimal role of volunteers |
| Sicilia et al., 2015 | S | U | 2 | I | Community NCD program | **Implementation problems**, namely the accessibility is not optimal |
| Suparto et al., 2015 | S | R | 1 | I | Community NCD program | **Education** can improve knowledge and skills can improve by training. I**mplementation problems**, i.e. capacity of CHWs was not optimal |
| Fuadah & Rahayu, 2018 | S | R | 2 | I | Community NCD program | **Implementation problems,** i.e. awareness of the community-based program |
| Astuti et al., 2016 | G | R | 3 | I | Community NCD program | **Implementation problems** i.e. incomprehensive and minimal training of volunteers, lack of equipment, lack of public awareness |
| Purdiyani, 2016 | S | R | 1 | I | Community NCD program | **Gender** is coherent with utilization of a community program |
| Sofiatin & Roesli, 2017 | S | U | 2 | I | Integrated health post for NCDs in community | **Synergy** with primary health care increases participation in community-based interventions |
| Jayusman & Widiyarta, 2017 | S | R | 2 | I | Community program for NCDs | **CHWs** are effective in screening and health checkups when there is **synergy** with primary healthcare |
| Kurnia et al, 2017 | S | R | 1 | I | Community NCD program | **Access** is coherent with utilization of a community-based program |
| Sumarni & Witdiawati, 2018 | S | NC | 2 | I | Community NCD program | Health **education** is needed for CHWs, with **involvement** of healthcare staff |
| Indriawati & Usman 2018 | S | R | 2 | I | Education and assistance to community in early detection and screening of NCDs | **CHWs** can effectively do activities such as screening and detection when **trained** |
| Dhippayom et al., 2012 | S | U | 2 | T | The Diabetes Prevention Program in community pharmacy using a diabetes risk prediction tool | **Lifestyle advice** from a community pharmacist can increase treatment adherence of diabetes patients, using a risk prediction, which is effective when combined with **referral** and **education** services |
| Ounnapiruk et al., 2014 | S | U | 2 | T | Group sessions and FGB to empower patients to motivate eat healthy diet, medication, and exercise | Community-based interventions aiming at empowerment are effective when organized in **groups** |
| Hanh, 2017 | G | R | 3 | V | Intervention to improve blood pressure monitoring and treatment adherence | Community interventions are effective when organized in **groups**, because of encouragement |
| Long, 2015 | G | R | 3 | V | Group-based education on hypertension prevention and treatment and home-based counselling | Community-based intervention are most effective when organized in **groups** |
| Aekplakorn et al., 2019 | S | NC | 1 | T | Lifestyle modification program through participatory group activities | Organizing community-based interventions in **groups** is effective for diabetes lifestyle |

^*^Type of literature: grey (G) literature or scientific (S) literature

^**^Type of area the study was performed: urban (U), rural (R) or not clear (NC)

^***^ Level of Quality in in which 1=high quality, 2=moderate quality and 3=weak quality

^****^ In which country the study was performed, in which C=Cambodia, I=Indonesia, M=Malaysia, P=The Philippines, T=Thailand, V=Vietnam, SEA=Southeast Asian Countries
